# Novel chloroquinoline derivatives incorporating biologically active benzenesulfonamide moiety: synthesis, cytotoxic activity and molecular docking

**DOI:** 10.1186/s13065-016-0164-1

**Published:** 2016-04-05

**Authors:** Mostafa M. Ghorab, Mansour S. Alsaid, Mohammed S. Al-Dosari, Yassin M. Nissan, Abdullah A. Al-Mishari

**Affiliations:** Department of Pharmacognosy, College of Pharmacy, King Saud University, P.O. Box 2457, Riyadh, 11451 Saudi Arabia; Department of Drug Radiation Research, National Center for Radiation Research and Technology, Nasr City, Cairo, 113701 Egypt; Department of Pharmaceutical Chemistry, Faculty of Pharmacy, Cairo University, Cairo, Egypt; Medicinal, Aromatic and Poisonous Plants Research Center (MAPPRC), College of Pharmacy, King Saud University, P.O. Box 2457, Riyadh, 11451 Saudi Arabia

**Keywords:** Chloroquinolines, Benzenesulfonamides, Anticancer activities

## Abstract

**Background:**

Quinoline derivatives have diverse biological activities including anticancer activity. On the other hand, many sulfonamide derivatives exhibited good cytotoxic activity. Hybrids of both moieties may present novel anticancer agents.

**Results:**

Chloroquinoline incorporating a biologically active benzene-sulfonamide moieties **5**–**21** and diarylsulfone derivatives **22** and **23** were prepared using (*E*)-1-(4-((*E*)-7-chloro-1-methylquinolin-4(1*H*)-ylideneamino)phenyl)-3-(dimethyl-amino)prop-2-en-1-one **4** as strategic starting material. The structure of the newly synthesized compounds were confirmed by elemental analyses and spectral data. Compound **4** was confirmed by X-ray crystallographic analysis. The prepared compounds were evaluated for their anticancer activity against Lung, HeLa, Colorectal and breast cancer cell lines. Compounds **2**, **4**, **7**, **11**, **14** and **17** showed better or comparable activity to 2′, 7′-dichlorofluorescein (DCF) as reference drug. Molecular docking of the active compounds on the active site of PI3K enzyme was performed in order to explore the binding mode of the newly synthesized compounds.

**Conclusion:**

Compounds **2**, **4**, **7**, **11**, **14** and **17** are novel quinoline derivatives that may represent good candidates for further evaluations as anticancer agents. The mechanism of action of these compounds could be through inhibition of PI3K enzyme.

**Graphical abstract:**
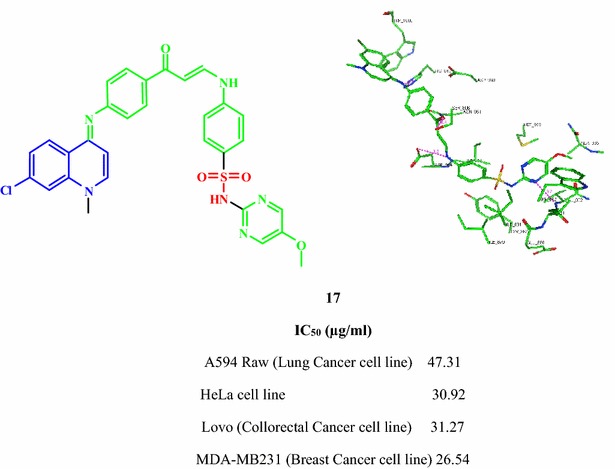
Compound **17** on the active site of PI3K

## Background

Quinoline scaffold has been broadly distributed in sundry natural and synthetic compounds with multipurpose biological activities [1–3]. The antitumor activity of the quinoline derivatives for instance camptothecin [4], luotonin [5], ascididemin [6], TAS-103 **A** that displayed IC_50_ value of: 0.0030–0.23 microM hostile to various cell lines [7], CIL-102 **B** that unveiled IC_50_ value of: 0.31–2.69 microM hostile to countless cell lines [8], cryptolepin [9] and indolo[2,3-b]quinolines [10] has been described. Numerous mechanisms of action were optional for such action among them was the strong suppression of E2F1 that inhibits growth by thwarting cell cycle progression and fasters differentiation by creating a permissive environment for cell distinction [11]. Chloroquinolines were valuable in sundry cancer sorts remarkably, breast cancer with high aptitude to induce apoptosis [12]. Heterocyclic sulfonamides have publicized good anticancer bustle with diversity of mechanisms embracing cell cycle perturbation at G1 phase, disruption of microtubules assembly and the eminent carbonic anhydrase inhibition activity with selectivity to the tumor allied isoforms hCA IX and hCA XII [13–17]. Merging quinoline scaffold with the biologically active benzene-sulfonamide moiety has received immense attention as PI3K inhibitor which is an vital enzyme regulatory signal transduction [16, 18–20]. Freshly, diaryl sulfones that were prepared from Dapson have shown respectable cytotoxic activity on breast cancer cell line [21]. Based on the aforementioned and as a continuation for our effort to synthesize a novel anticancer agents [18–25], we have prepared novel quinolone-sulfonamide and diarylsulfone derivatives. Prepared compounds were subjected to cytotoxic assay on lung, hela, colorectal and breast cancer cell lines. Likewise, “the highest active compounds were docked on the active site of PI3K enzyme” to recommend their binding mode in a trial to explore their mechanism of action expecting to reach innovative anticancer agents. 

## Results and discussion

### Chemistry

**Fig. 1:**
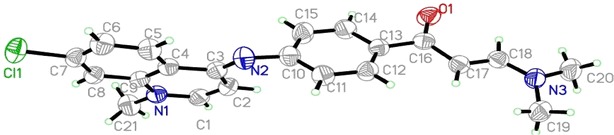
ORTEP diagram of the title compound **4** drawn at 40 % ellipsoids for non-hydrogen atoms

**Fig. 2:**
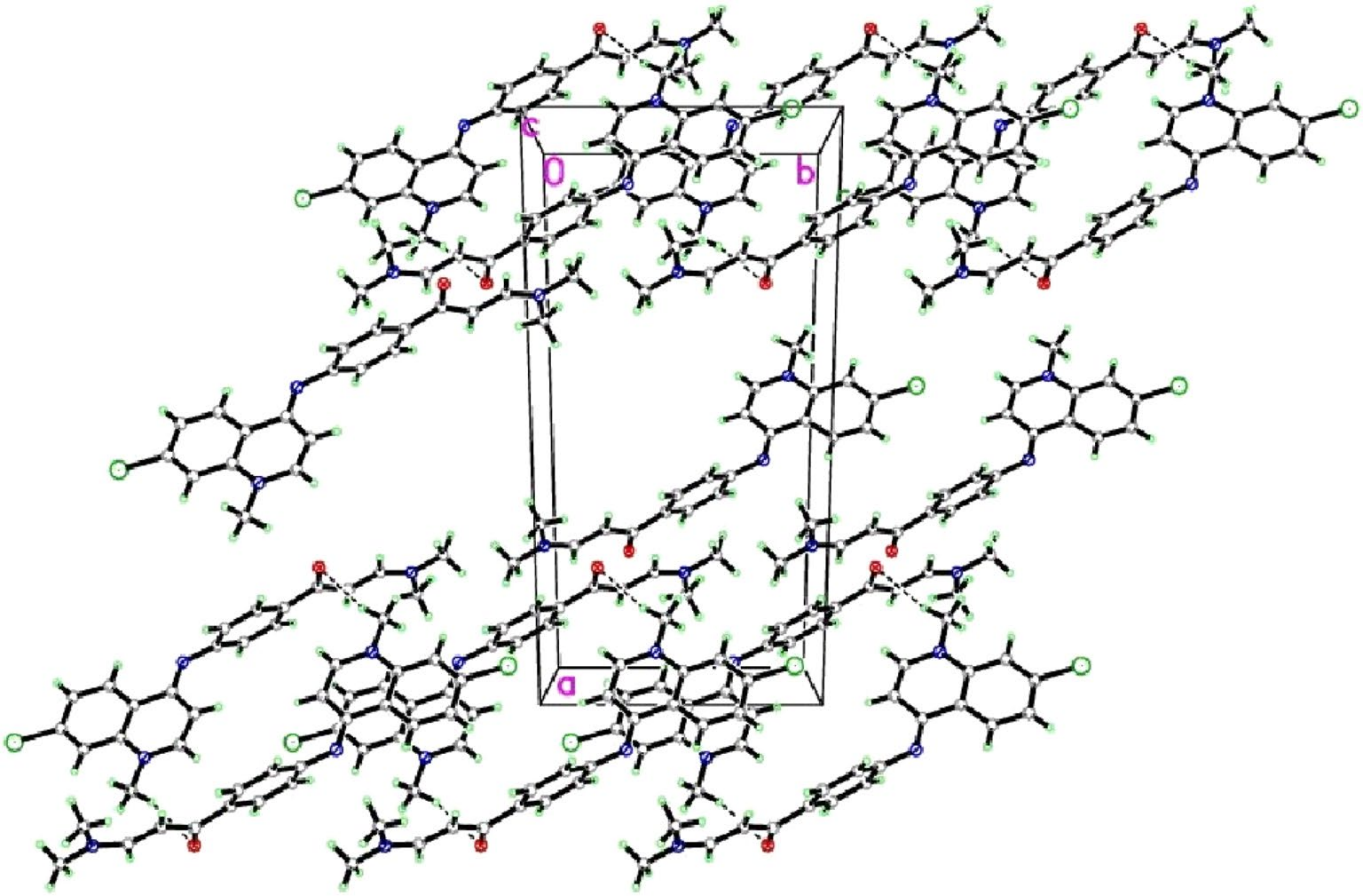
Crystal packing of compound **4** showing the intermolecular hydrogen bonds

**Scheme 1:**
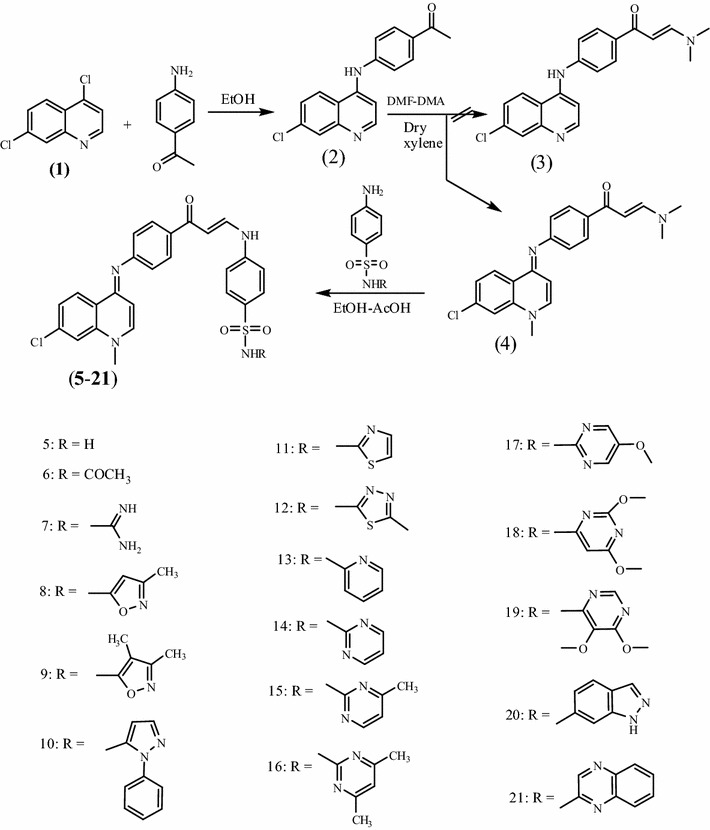
Synthetic pathways for compounds **5**–**21**

**Scheme 2:**
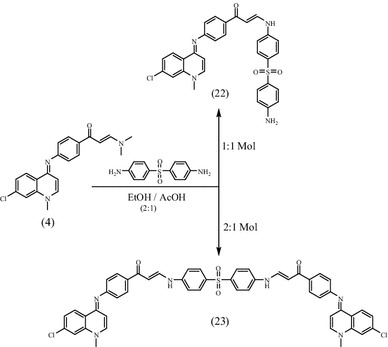
Synthetic pathways for compounds **22** and **23**

The ambition of this effort was to prepare a new series of chloroquinolines carrying biologically active benzene-sulfonamide moieties and to assess their anticancer activity. Thus, interaction of **2** [26] with dimethylformamide-dimethylacetal (DMF-DMA) in dry xylene yielded the unexpected **4** instead of expected **3**. “The structural assignments to synthesized compounds were based on their physico-chemical characteristics and spectroscopic (FT-IR, ^1^H-NMR, ^13^C-NMR, and mass spectroscopy) investigations”. Structure of **4** was confirmed by X-ray crystallographic analysis [27] (Figs. 1, 2). IR of **4** revealed the disappearance of NH band and presence of absorption bands for (aromatic), (aliphatic), (CO), (CN), (CCl). ^1^H-NMR showed the presence of a singlet at 2.4 ppm attributed to N-(CH_3_)_2_, singlet at 3.4 ppm assigned to N-CH_3_, two doublet at 5.4, 6.5 ppm for CH = CH of quinolone ring, two doublet at 6.1,7.4 ppm assigned to CH = CH group. Enaminones are highly reactive intermediates extensively used for the preparation of heterocyclic derivatives. Thus, treatment of 4-(7-chloro-1-methylquinolin-4-(1*H*)-ylideneamino) phenyl-3-(dimethyl-amino)-prop-2-en-1-one **4** with sulfonamide derivatives in refluxing ethanol/acetic acid mixture (2:1) afforded the sulfonamide derivatives **5**–**21** (Scheme 1). “Structures of the latter products were assigned on the basis of their analytical and spectral data”. ^1^H NMR of **5**–**21** support the assumption that these structures were in E-form and not in Z form, while the coupling constant of doublet signals for olefinic protons was equal to 6.1–7.7 Hz. IR of the reaction products showed in each case three absorption bands for 2NH functions in the 3446–3143 cm^−1^ region, in addition to carbonyl functions 1654–1635 cm^−1^ region and CCl functions 883–763 cm^−1^ (Scheme 1). ^1^H-NMR of **5** showed singlet at 12.0 ppm assigned to NH group, while ^13^C NMR revealed singlet at 189.3 ppm for CO group. ^1^H-NMR of **6** exhibited singlet at 2.0 ppm according to COCH_3_ group.^1^H-NMR of **7** revealed singlet at 9.4 ppm for NH group. ^1^H-NMR of **8** showed singlet at 2.3 ppm for CH_3_ group, while ^1^H NMR of **9** exhibited two signals at 1.9, 2.6 assigned to 2CH_3_ groups. ^1^H NMR of **10** revealed two signals at 10.2, 12.0 ppm assigned to NH, SO_2_NH groups. ^1^H-NMR of **11** exhibited two signals at 6.6, 6.8 ppm for CH = CH of thiazole ring. ^1^H-NMR of **12** exhibited singlet at 2.4 ppm for CH_3_ of thiadiazole ring. ^13^C NMR of **13** showed signal at 186.6 ppm due to CO group. ^1^H-NMR of **15** exhibited singlet at 2.3 ppm for CH_3_ of pyrimidine ring. ^1^H-NMR of **16** revealed singlet at 2.2 ppm for 2CH_3_ of pyrimidine ring. ^1^H-NMR of compound **17** exhibited singlet at 3.9 ppm for OCH_3_ group. ^1^H-NMR of **18** showed singlet at 3.7 ppm assigned to 2OCH_3_ groups, while ^1^H NMR of **19** exhibited two signals at 3.6, 3.8 ppm attributed to 2OCH_3_ groups. ^1^H NMR of **20** revealed singlet at 12.0 according to NH group of indazole ring. ^13^C-NMR of **21** showed singlet at 186.7 ppm for CO group. Interaction of **4** with Dapson in molar ratio (1:1 mol) afforded the mono compound **22**, while the bis-compound **23** was achieved in the same condition but in molar ratio (2:1 mol). Compounds **22** and **23** were confirmed by microanalyses, IR, ^1^H-NMR, ^13^C-NMR and mass spectral data. IR of **22** revealed the characteristic bands at 3446, 3348, 3213 cm^−1^ (NH_2_, NH), 1635 cm^−1^ (CO), 1591 cm^−1^ (CN), 1369, 1180 cm^−1^ (SO_2_), 821 cm^−1^ (CCl). ^1^H-NMR of **22** exhibited signals at 3.4 ppm corresponding to N-CH_3_ group, 5.9 ppm due to NH_2_ group, two doublet at 6.1, 7.4 ppm for 2 CH quinoline, two doublet at 6.5, 6.6 ppm assigned to CH = CH groups, singlet at 12.0 NH. ^13^C-NMR of **22** showed singlet at 186.6 ppm attributed to (CO) group. Mass of **22** revealed a molecular ion peak m/z at 569 [M^+^] (19.87) with a base peak appeared at 90 (100). IR of **23** showed a characteristic bands at 3143 cm^−1^ (2NH), 1635 cm^−1^ (2CO), 1570 cm^−1^ (2CN), 1375, 1180 cm^−1^ (SO_2_), 819 cm^−1^ (2CCl). ^1^H-NMR of **23** revealed signals at 3.4 ppm for N-CH_3_, two doublets at 6.2, 7.3 ppm due to 4CH quinoline, two doublets at 6.6, 7.2 assigned to 2CH = CH, two singlet’s at 9.3, 12.0 for 2NH groups. ^13^C-NMR of **23** revealed singlet at 186.7 ppm for (2CO) groups. Mass of **23** showed a molecular ion peak m/z at 889 [M^+^] (6.48) with a base peak appeared at 272 (100) (Scheme 2).

### In vitro cytotoxic screening

The newly synthesized compounds were evaluated for their in vitro cytotoxic activity against human lung (A549-Raw), hela, colorectal (lovo) and breast (MDA-MB231) cancer cell lines and 2′,7′-dichlorofluorescein (DCF) was used as the reference drug in this study. The relationship between surviving fraction and drug concentration was plotted to obtain the survival curve of cancer cell lines. The response parameter calculated was the IC_50_ value, which corresponds to the concentration required for 50 % inhibition of cell viability. Table 1 shows the in vitro cytotoxic activity of the newly synthesized compounds. In a closer look to Table 1, we can see that compounds **1**, **2**, **4**, **7**, **11**, **14** and **17** were active towards all the tested cell line while the rest of compounds were inactive. Regarding the activity towards lung cancer cell line (A549-Raw), all the aforementioned compounds were more active than DCF as reference drug. Compound **2** was the most active compound with IC_50_ value of 44.34 μg/ml. For Hela cancer cell line, the same compounds were active. Compounds **7** and **17** were more active than DCF and compound **17** was the most active compound with IC_50_ value of 30.92 μg/ml. In case of lovo cancer cell line, all seven compounds were more active than DCF. Compound **2** was the most active compound with IC_50_ value of 28.82 μg/ml. Finally, the activity towards breast cancer cell line (MDA-MB231) was better than that of DCF for the aforementioned compounds except for compound **14**. Compound **17** again was the most active compound with IC_50_ value of 26.54 μg/ml. In the light of biological results, we can see that the 4,7-dichloroquinoline **1** showed moderate anticancer activity that were enhanced upon converting it to 1-(4-(7-chloloquinoline-4-ylamino) phenyl)ethanone **2**. The activity still exists upon preparation of (*E*)-1-(4-((*E*)-7-chloro-1-methylquinolin-4(1*H*)-ylideneamino) phenyl)-3-(dimethylamino) prop-2-en-1-one **4**. Further preparation of the sulfonamide derivatives **5**–**21** using various sulfa drugs only succeeded to obtain active derivatives with the guanidine derivative **7**, the thiazole derivative **11**, the pyrimidine derivative **14** and the 5-methoxypyrimidine derivative **17**. Combination with diaryl sulfone moieties as in compounds **22** and **23** did not yield active compounds.

**Table 1:** In vitro anticancer screening of the newly synthesized compounds against four cancer cell lines

Compound no.	A549-Raw (lung cancer cells)	Hela cells	Lovo (colorectal cancer cells)	MDA-MB231 (breast cancer cells)
IC_50_ (µg/ml)				
**1**	68.74	84.20	84.26	77.78
**2**	44.34	56.32	28.82	38.83
**4**	76.73	88.66	104.78	72.85
**5**	na	na	na	na
**6**	na	na	na	na
**7**	91.0	51.58	39.09	55.58
**8**	na	na	na	na
**9**	na	na	na	na
**10**	na	na	na	na
**11**	97.27	91.74	81.89	111.90
**12**	na	na	na	na
**13**	na	na	na	na
**14**	96.45	94.63	93.72	115.11
**15**	na	na	na	na
**16**	na	na	na	na
**17**	47.31	30.92	31.27	26.54
**18**	na	na	na	na
**19**	na	na	na	na
**20**	na	na	na	na
**21**	na	na	na	na
**22**	na	na	na	na
**23**	na	na	na	na
**DCF**	124.87	54.07	114.12	113.94

*na* not active

### Molecular docking

**Fig. 3:**
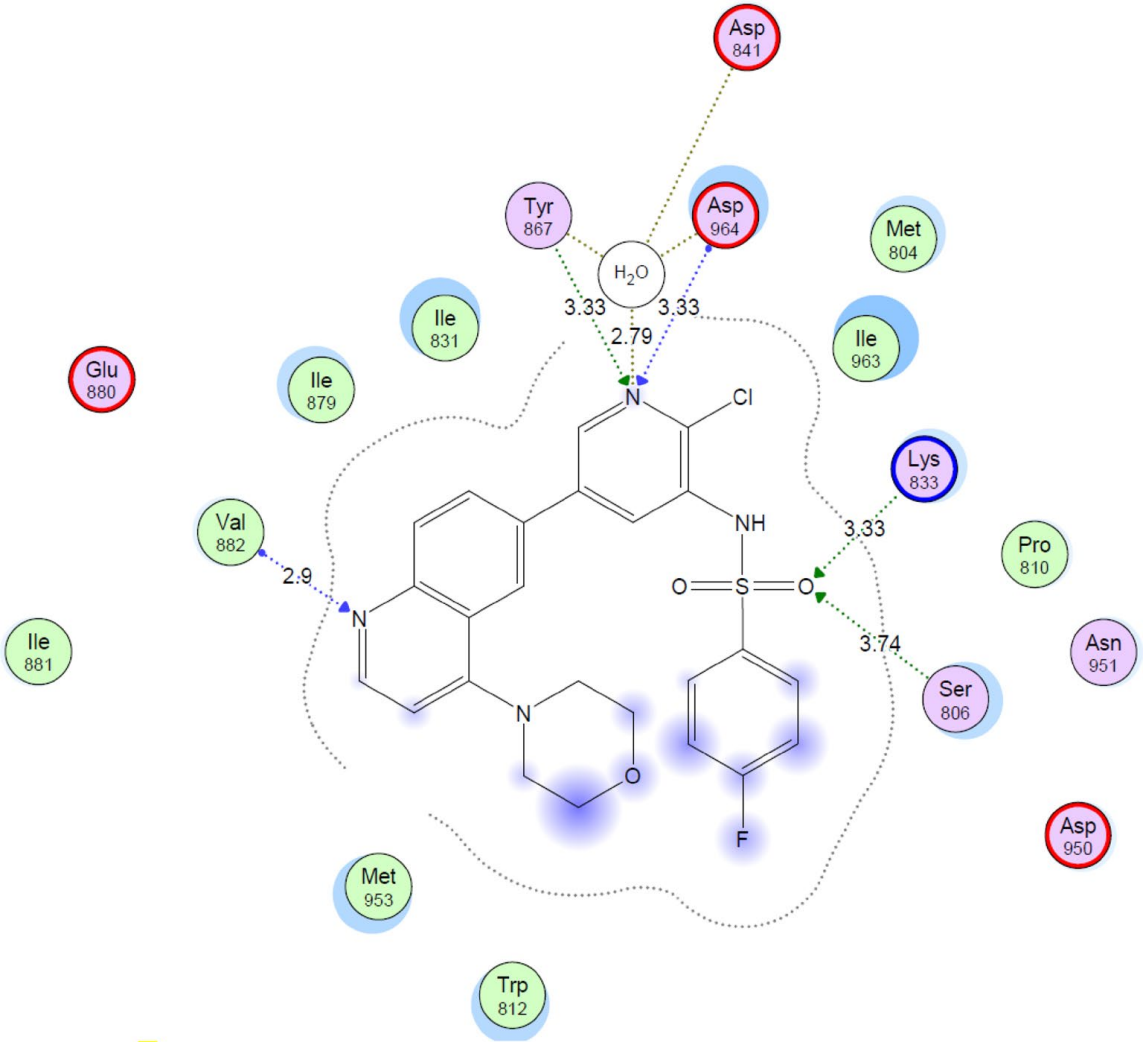
Co-crystallized quinoline ligand on the active site of phosphoinisitol kinase (PI3K)

**Fig. 4:**
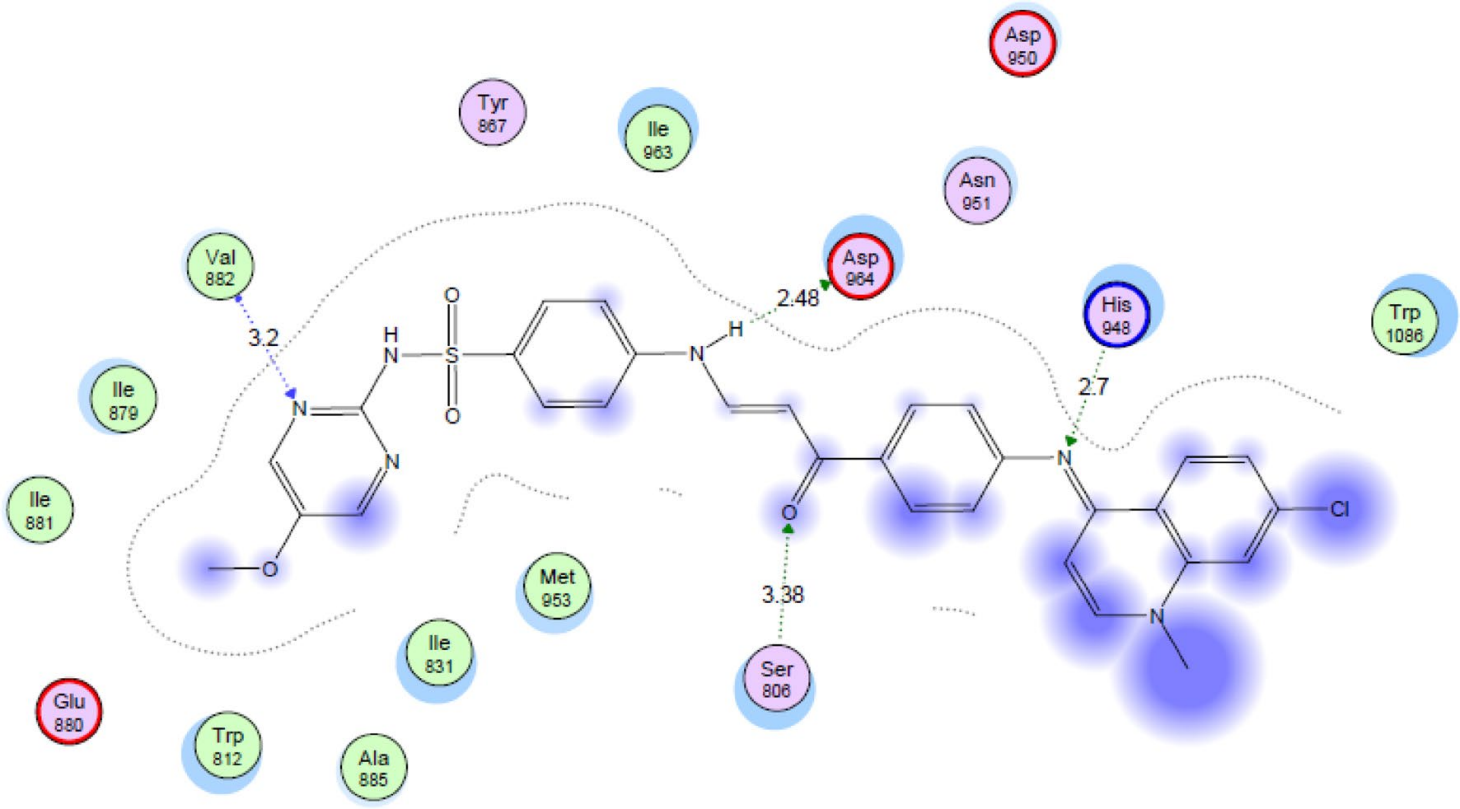
2D interactions of compound **17** on the active site ofphosphoinisitol kinase (PI3K)

**Fig. 5:**
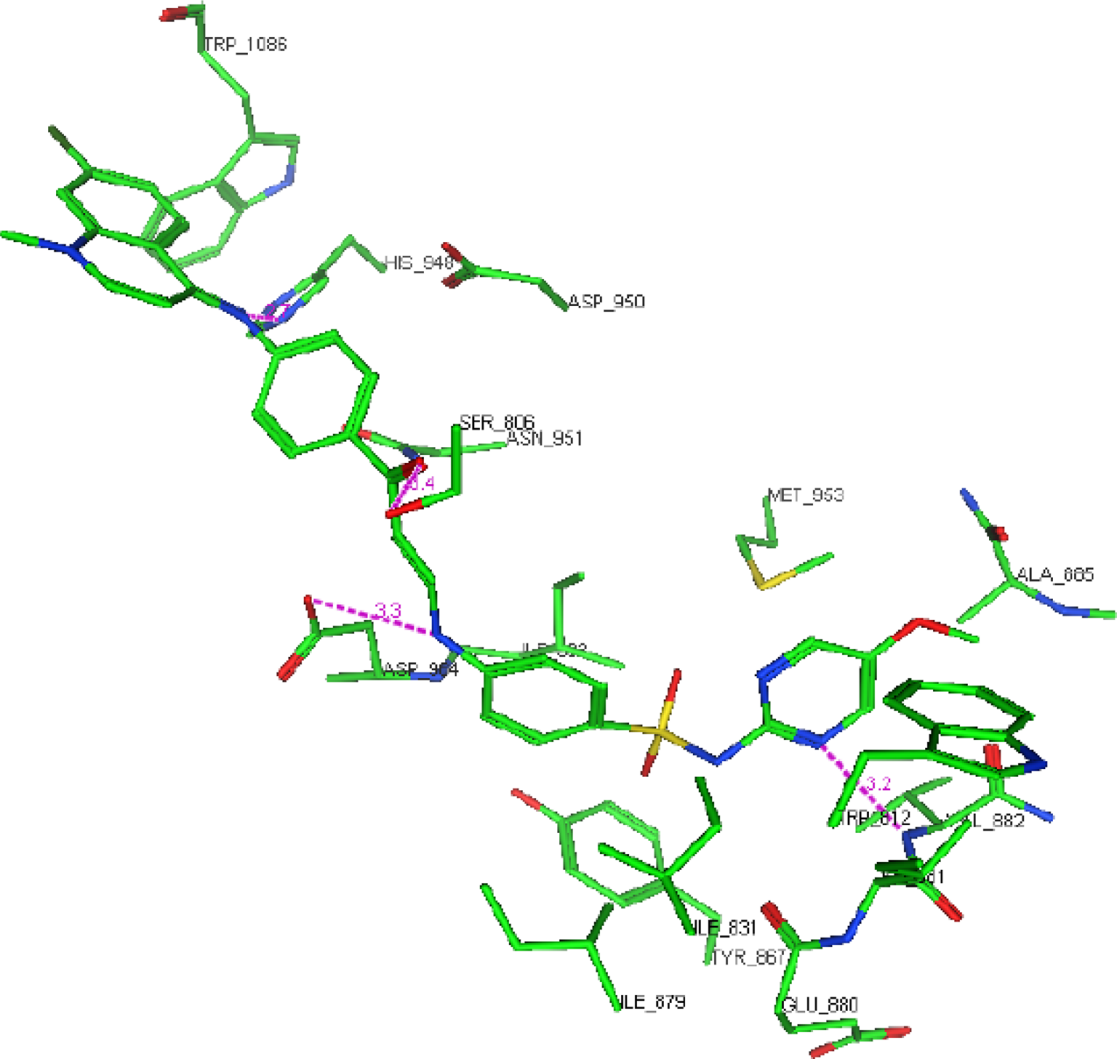
3D interactions of compound **17** on the active site of phosphoinisitol kinase (PI3K)

Phosphoinositide 3-kinases (PI3K) comprises an important class of enzymes that phosphorylates the 3 hydroxyl group of inisitol and play a major role in signal transduction through the cell cycle. Targeting PI3K by inhibitors has become a well-known strategy in seeking for new anticancer agents [28]. Quinolinesulfonamide derivatives were reported to express good inhibitory activity on PI3K enzyme [16]. In our present investigation and in a trial to suggest the mechanism of action of the active compounds, molecular docking of compounds **1**, **2**, **4**, **7**, **11**, **14** and **17** was performed on the active site of PI3K to explore their binding modes to amino acids of the active site of the enzyme. The protein data bank file (PDB: 3S2A) was selected for this purpose. The file contains PI3K enzyme co-crystallized with a quinoline ligand. All docking procedures were achieved by MOE (Molecular Operating Environment) software 10.2008 provided by chemical computing group, Canada. Docking on the active site of PI3K enzyme was performed for all synthesized compounds. Docking protocol was verified by redocking of the cocrystallized ligand in the vicinity of the active site of the enzyme with energy score (S) = −29.8249 kcal/mol and root mean standard deviation (RMSD) = 1.9094 (Fig. 3). The quinoline ligand interacts with the active site of PI3K by six interactions: Val 882 with a hydrogen bond of 2.90 Å, Tyr 867 with a hydrogen bond of 3.33 Å, Asp 864 with a hydrogen bond of 3.33 Å, Lys 833 with a hydrogen bond of 3.33 Å, Ser 806 with a hydrogen bond of 3.74 Å and Asp 841 with a hydrogen bond of 2.79 Å through a water molecule. All the docked compounds were fit in the active site of enzyme. Energy scores (S) as well as amino acids interactions were listed in Table 2. The best docking score was achieved by compound **17** with a value = −27.1666 kcal/mol. Compound **17** interacted with Val 822 with a hydrogen bond of 3.20 Å, with Asp 964 with a hydrogen bond of 2.48 Å, with Ser 806 with a hydrogen bond of 3.38 Å and finally with His 984 with a hydrogen bond of 2.70 Å (Figs. 4, 5).

**Table 2:** Binding scores and amino acid interactions of the docked compounds on the active site of phosphoinisitol kinase (PI3K)

Compound no.	S Kcal/Mol	Amino acid interactions	Interacting groups	Type of interaction	H bond length Å
**1**	−15.0154	Val 882	N-quinoline	H-bond (acceptor)	2.87
**2**	−19.6829	Val 882	N-quinoline	H-bond (acceptor)	3.5
		Lys 802	CO	H-bond (acceptor)	2.42
		Lys 890	Phenyl	Arene-cation	
**4**	−15.8363	Val 882	CO	H-bond (acceptor)	2.58
**7**	−15.2630	Val 882	CO	H-bond (acceptor)	2.95
		Asp 964	C = NH	H-bond (donor)	1.48
		Lys 890	Phenyl	Arene-cation	
**11**	−14.8730	Val 882	CO	H-bond (acceptor)	3.15
		Lys 883	SO_2_	H-bond (acceptor)	2.97
		Ala 885	NH	H-bond (donor)	1.74
		Glu 814	SO_2_NH	H-bond (donor)	1.34
**14**	−22.7755	Val 882	CO	H-bond (acceptor)	2.86
		Lys 883	SO_2_	H-bond (acceptor)	2.80
		Lys 883	N-pyrimidine	H-bond (acceptor)	3.00
		Lys 890	Phenyl	Arene-cation	
**17**	−27.1666	Val 882	N-pyrimidine	H-bond (acceptor)	3.20
		Asp 964	NH	H-bond (donor)	2.48
		Ser 806	CO	H-bond (acceptor)	3.38
		His 948	CN	H-bond (acceptor)	2.70

## Experimental

### Chemistry

Melting points (uncorrected) were determined in open capillary on a Gallen Kamp melting point apparatus (Sanyo Gallen Kamp, UK). Precoated silica gel plates *(Kieselgel* 0.25 mm, 60 F254, Merck, Germany) were used for thin layer chromatography. A developing solvent system of chloroform/methanol (8:2) was used and the spots were detected by ultraviolet light. IR spectra (KBr disc) were recorded using an FT-IR spectrophotometer (Perkin Elmer, USA). ^1^H-NMR spectra were scanned on an NMR spectrophotometer (Bruker AXS Inc., Switzerland), operating at 500 MHz for ^1^H- and 125.76 MHz for ^13^C. Chemical shifts are expressed in δ-values (ppm) relative to TMS as an internal standard, using DMSO-*d*_*6*_ as a solvent. Elemental analyses were done on a model 2400 CHNSO analyser (Perkin Elmer, USA). All the values were within ±0.4 % of the theoretical values. All reagents used were of AR grads.

### (*E*)-1-(4-((*E*)-7-chloro-1-methylquinolin-4(1*H*)-ylideneamino)phenyl)-3-(dimethylam-ino)prop-2-en-1-one (4)

1-(4-(7-chloroquinoline-4-ylamino)phenyl)ethanone **2** (2.97 g, 0.01 mol) and dimethylformamide-dimethylacetal (1.19 g, 0.01 mol) was added into dry xylene (30 mL). Reaction was refluxed for 10 h, and the solid product recrystallized from ethanol to give **4**.

Yield, 89 %; m.p.268.1 °C. IR: 3100 (arom.), 2966, 2856 (aliph.), 1696 (CO), 1618 (CN), 776 (CCl).). ^1^HNMR: 2.4 [s, 3H, N(CH_3_)_2_], 3.6 [s, 1H, N-CH_3_], 5.4, 6.5 [2d, 2H, CH = CH quinoline, *J* = 7.1, 7.3 Hz], 6.1,7.4 [2d, 2H, CH = CH, *J* = 7.5, 7.4 Hz], 6.9–7.6 [m, 3H, Ar–H]. ^13^CNMR: 36.3, 44.5 (2), 91.5, 114.6, 115.3, 116.9, 121.4 (2), 131.7, 132.8 (2), 133.0, 135.9, 136.6, 141.4, 146.2, 152.5, 161.4, 166.4, 191.3. MS m/z (%): 365 (M^+^) (2.84), 74 (100). Anal.Calcd. For C_21_H_20_ClN_3_O (365.86): C, 68.94; H, 5.51; N, 11.49. Found: C, 68.66; H, 5.22; N, 11.74.

### Synthesis of sulfonamide derivatives **5**–**21**

4-(7-chloro-1-methylquinolin-4-(1*H*)-ylideneamino) phenyl-3-(dimethylamino)-prop-2-en-1-one **4** (3.65 g, 0.01 mol) and sulfa-drugs (0.012 mol) was added into ethanol (10 mL) and acetic acid (5 mL). The mixture was refluxed for 18 h. The solid product formed was recrystallized from dioxane to give **5**–**21**.

### 4-(*E*)-3-(4-(*E*)-7-chloro-1-methylquinolin-4(1*H*)-ylideneamino) phenyl)-3-oxoprop-1-en-ylamino)benzenesulfonamide (5)

Yield, 88 %; m.p. 299.0 °C. IR: 3381, 3209 (NH_2_, NH), 3078 (arom.), 2937, 2869 (aliph.), 1635 (CO), 1593 (CN), 1373, 1182 (SO_2_), 867 (CCl). ^1^HNMR: 3.6 [s, 3H, N-CH_3_], 6.2, 7.3 [2d, 2H, 2CH quinoline, *J* = 7.2 Hz], 6.1, 7.6 [2d, 2H, CH = CH, *J* = 7.4 Hz], 7.7–8.6 [m, 13H, Ar–H + SO_2_NH_2_], 12.0 [s, 1H, NH]. ^13^CNMR: 40.5, 95.1, 99.8, 104.9 (2), 112.5, 115.4, 116.2, 119.5 (2), 125.8 (2), 127.9, 128.2 (2), 133.8, 137.6, 138.4, 143.1, 144.6, 146.7, 152.5, 172.5, 189.3. MS m/z (%): 492 (M^+^) (4.72), 91 (100). Anal. Calcd. For C_25_H_21_ClN_4_O_3_S (492.98): C, 60.91; H, 4. 29; N, 11.36. Found: C, 61.19; H, 4.52; N, 11.01.

### *N*-(4-(*E*)-3-(4-(*E*)-7-chloro-1-methylquinolin-4(1*H*)-ylideneamino)phenyl)-3-oxoprop-1-enylamino)phenylsulfonyl)acetamide(6)

Yield, 76 %; m.p. 310.0 °C. IR: 3367 (NH), 3066 (arom.), 2939, 2877 (aliph.), 1724, 1635 (2CO), 1593 (CN), 1369,1184 (SO_2_), 833 (CCl). ^1^HNMR: 2.0 [s, 3H, COCH_3_], 3.5 [s, 3H, N-CH_3_], 6.3, 7.3 [2d, 2H, 2CH quinoline, *J* = 7.4 Hz], 6.6, 7.6 [2d, 2H, CH = CH, *J* = 7.6 Hz], 7.7–8.6 [m, 12H, Ar–H + SO_2_NH], 12.0 [s, 1H, NH]. ^13^CNMR: 23.6, 40.5, 97.8, 101.3, 112.7(2), 115.1, 116.0, 119.5, 120.2 (2), 125.9 (2), 128.1, 129.5 (2), 130.2, 134.6, 142.8 (2), 144.5, 146.9, 150.0, 152.4, 163.1, 186.7, 189.6. MS m/z (%): 535 (M^+^) (9.36), 74 (100). Anal. Calcd. For C_27_H_23_ClN_4_O_4_S (535.01): C, 60.61; H, 4.33; N, 10.47. Found: C, 60.29; H, 4.59; N, 10.19.

### *N*-carbamimidoyl-4-(*E*)-3-(4-(*E*)-7-chloro-1-methylquinolin-4(1*H*)-ylideneamino)- phenyl)-3-oxoprop-1-enylamino)benzenesulfonamide (7)

Yield, 81 %; m.p. 146.6 °C. IR: 3431, 3336, 3209 (NH_2_, NH), 3100 (arom.), 2957, 2858 (aliph.), 1635 (CO), 1593 (CN), 1373, 1178 (SO_2_), 827 (CCl). ^1^HNMR: 3.4 [s, 3H, NCH_3_], 6.2, 7.6 [2d, 2H, 2CH quinoline, *J* = 7.3 Hz], 6.1, 7.4 [2d, 2H, CH = CH, *J* = 7.4 Hz], 7.7–8.6 [m, 13H, Ar–H + NH_2_], 9.4 [s, 1H, NH imino], 10.3, 12.0 [2s, 2H, NH + SO_2_NH]. ^13^CNMR: 40.5, 94.9, 99.4, 112.8 (2), 115.2, 116.1, 119.5, 120.2 (2), 125.8 (2), 127.8, 129.5 (2), 131.2, 133.8, 134.6, 138.0, 142.9, 144.8, 145.1, 158.2, 158.5, 172.8, 189.2. MS m/z (%): 535 (M^+^) (7.74), 76 (100). Anal. Calcd. For C_26_H_23_ClN_6_O_3_S (535.02): C, 58.37; H, 4. 33; N, 15.71. Found: C, 58.55; H, 4.09; N, 15.47.

### 4-(*E*)-3-(4-(*E*)-7-chloro-1-methylquinolin-4(1*H*)-ylideneamino) phenyl)-3-oxoprop-1-en-ylamino)-*N*-(3-methylisoxazol-5-yl)benzenesulfonamide (8)

Yield, 86 %; m.p. 192.5 °C. IR: 3446, 3215 (NH), 3088 (arom.), 2970, 2883 (aliph.), 1635 (CO), 1616 (CN), 1369,1159 (SO_2_), 821 (CCl). ^1^HNMR: 2.3 [s, 3H, CH_3_], 3.4 [s, 3H, NCH_3_], 6.1, 7.3 [2d, 2H, 2CH quinoline, *J* = 7.7 Hz], 6.6, 7.6 [2d, 2H, CH = CH, *J* = 7.4 Hz], 6.7 [s, 1H, CH isoxazole], 7.7–8.5 [m, 12H, Ar–H + SO_2_NH], 12.0 [s,1H, NH]. ^13^CNMR: 12.4, 40.5, 95.5, 100.4, 104.7, 113.0 (2), 115.5, 116.3, 119.5, 120.1 (2), 125.8, 129.2 (2), 132.9 (2), 133.7, 134.6, 142.8, 144.9, 145.2, 146.8, 147.4, 153.7, 154.3, 158.5, 170.5, 186.9. MS m/z (%): 574 (M^+^) (1.62), 58 (100). Anal. Calcd. For C_29_H_24_ClN_5_O_4_S (574.05): C, 60.68; H, 4. 21; N, 12.20. Found: C, 60.39; H, 4.54; N, 12.49.

### 4-(*E*)-3-(4-(*E*)-7-chloro-1-methylquinolin-4(1*H*)-ylideneamino) phenyl)-3-oxoprop-1-en-ylamino)-*N*- (3,4-dimethylisoxazol-5-yl)benzenesulfonamide (9)

Yield, 77 %; m.p. 212.1 °C. IR: 3381, 3230 (NH), 3099 (arom.), 2926, 2819, 2763 (aliph.), 1635 (CO), 1589 (CN), 1373, 1180 (SO_2_), 810 (CCl). H^1^ NMR: 1.9, 2.6 [2s, 6H, 2CH_3_], 3.4 [s, 3H, NCH_3_], 6.2, 7.3 [2d, 2H, 2CH quinoline, *J* = 7.6 Hz], 6.6, 7.5 [2d, 2H, CH = CH, *J* = 7.5 Hz], 7.6–8.6 [m, 11H, Ar–H], 10.4, 12.0 [2s,2H, NH +SO_2_NH]. ^13^CNMR: 6.4, 10.8, 40.5, 95.5, 100.3, 102.9, 104.4 (2), 115.5, 116.4, 119.2, 120.7 (2), 126.1, 127.3 (2), 129.5 (2), 133.6, 134.1, 135.2, 142.9, 144.4, 145.4, 147.7, 157.4, 157.9, 161.5, 172.5, 189.3. MS m/z (%): 588 (M^+^) (11.22), 55 (100). Anal. Calcd. For C_30_H_26_ClN_5_O_4_S (588.08): C, 61.27; H, 4. 46; N, 11.91. Found: C, 61.01; H, 4.17; N, 11.64.

### 4-(*E*)-3-(4-(*E*)-7-chloro-1-methylquinolin-4(1*H*)-ylideneamino) phenyl)-3-oxoprop-1-en-ylamino)-*N*-(1-phenyl-1H-pyrazol-5-yl)benzenesulfonamide (10)

Yield, 80 %; m.p. 94.3 °C. IR: 3417, 3230 (NH), 3064 (arom.), 2966, 2827 (aliph.), 1635 (CO), 1591 (CN), 1373, 1180 (SO_2_), 763 (CCl). ^1^HNMR: 3.4 [s, 3H, NCH_3_], 6.2, 7.5 [2d, 2H, 2CH quinoline, *J* = 7.5 Hz], 6.5, 7.2 [2d, 2H, CH = CH, *J* = 7.7 Hz], 7.8–8.6 [m, 18H, Ar–H], 10.2, 12.0 [2s, 2H, NH +SO_2_NH]. ^13^CNMR: 40.5, 97.3, 100.0, 103.5, 111.6 (2), 113.0, 116.2, 118.6, 123.7 (2), 124.7 (2), 125.1, 129.0 (2), 129.1, 129.2 (2), 129.3 (2), 129.4, 129.5, 135.1, 136.2, 137.7, 138.9, 140.2, 142.7, 144.3, 146.1, 156.8, 172.4, 186.8. MS m/z (%): 635 (M^+^) (4.43), 103 (100). Anal. Calcd. For C_34_H_27_ClN_6_O_3_S (635.13): C, 64.30; H, 4. 28; N, 13.23. Found: C, 64.56; H, 4.52; N, 13.49.

### 4-(*E*)-3-(4-(*E*)-7-chloro-1-methylquinolin-4(1*H*)-ylideneamino) phenyl)-3-oxoprop-1-en-ylamino)-*N*-(thiazol-2-yl) benzenesulfonamide (11)

Yield, 69 %; m.p. 172.7 °C. IR: 3341, 3219 (NH), 3101 (arom.), 2937, 2869 (aliph.), 1635 (CO), 1589 (CN), 1373, 1180 (SO_2_), 773 (CCl). ^1^HNMR): 3.4 [s, 3H, N-CH_3_], 5.8, 7.6 [2d, 2H, 2CH quinoline, *J* = 7.0 Hz], 6.2, 7.2 [2d, 2H, CH = CH, *J* = 7.3 Hz], 6.6, 6.8 [2d, 2CH thiazole, *J* = 7.9 Hz], 7.7–8.6 [m, 11H, Ar–H], 10.2, 12.0 [2s, 2H, NH + SO_2_NH]. ^13^CNMR: 40.5, 95.1, 99.8, 108.5, 112.9(2), 115.3, 116.2, 119.5, 120.1 (2), 125.9, 128.3 (2), 129.5 (2), 133.0, 134.6, 135.7, 136.9, 143.0, 144.6, 145.1, 146.9, 152.6, 168.4, 172.5, 186.6. MS m/z (%): 576 (M^+^) (8.99), 101 (100). Anal. Calcd. For C_28_H_22_ClN_5_O_3_S_2_ (576.09): C, 58.38; H, 3.85; N, 12.16. Found: C, 58.23; H, 4.11; N, 12.46.

### 4-(*E*)-3-(4-(*E*)-7-chloro-1-methylquinolin-4(1*H*)-ylideneamino) phenyl)-3-oxoprop-1-en-ylamino)-*N*-(5-methyl-1,3,4-thiadiazol-2-yl)benzenesulfonamide (12)

Yield, 82 %; m.p. 304.3 °C. IR: 3246, 3115 (NH), 3088 (arom.), 2937, 2859 (aliph.), 1635 (CO), 1589 (CN), 1383, 1182 (SO_2_), 769 (CCl). ^1^HNMR: 2.4 [s, 3H, CH_3_ thiadiazole], 3.4 [s, 3H, N-CH_3_], 6.2, 7.6 [2d, 2H, 2CH quinoline, *J* = 7.6 Hz], 6.6, 7.2 [2d, 2H, CH = CH, *J* = 7.8 Hz], 7.7–8.5 [m, 11H, Ar–H], 10.3, 12.0 [2s, 2H, NH + SO_2_NH]. ^13^CNMR: 16.4, 40.5, 95.2, 99.9, 115.4 (2), 116.3, 120.2, 120.4, 125.2 (2), 127.9, 128.2 (2), 129.5 (2), 133.1, 134.8, 135.3, 143.0, 143.8, 144.6, 144.8, 152.1, 154.7, 168.3, 172.4, 189.3. MS m/z (%): 591 (M^+^) (25.7), 178 (100). Anal. Calcd. For C_28_H_23_ClN_6_O_3_S_2_ (591.10): C, 56.89; H, 3.92; N, 14.22. Found: C, 56.59; H, 3.68; N, 14.49.

### 4-((*E*)-3-(4-((*E*)-7-chloro-1-methylquinolin-4(1*H*)-ylideneamino)phenyl)-3-oxoprop-1-enylamino)-*N*-(pyridin-2-yl)benzenesulfonamide (13)

Yield, 91 %; m.p. 177.1 °C. IR: 3323, 3219 (NH), 3080 (arom.), 2939, 2849 (aliph.), 1654 (CO), 1596 (CN), 1375, 1178 (SO_2_), 773 (CCl). ^1^HNMR: 3.4 [s, 3H, NCH_3_], 6.2, 7.6 [2d, 2H, 2CH quinoline, *J* = 7.6 Hz], 6.6, 7.3 [2d, 2H, CH = CH, *J* = 7.1 Hz], 7.7–8.6 [m, 15H, Ar–H],10.3, 12.0 [2s, 2H, NH +SO_2_NH]. ^13^CNMR: 40.5, 95.3, 100.0, 104.9, 112.9 (2), 113.7, 115.3, 116.4, 119.5, 120.2 (2), 128.2, 129.5 (2), 132.9 (2), 133.7, 134.4, 135.7, 140.3, 142.9, 143.9, 144.6, 145.2, 146.7, 152.4, 153.4, 172.5, 186.6. MS m/z (%): 570 (M^+^) (18.2), 79 (100). Anal. Calcd. For C_30_H_24_ClN_5_O_3_S (570.06): C, 63.21; H, 4. 24; N, 12. 29. Found: C, 63.47; H, 4.52; N, 12.55.

### 4-((*E*)-3-(4-((*E*)-7-chloro-1-methylquinolin-4(1*H*)-ylideneamino)phenyl)-3-oxoprop-1-enylamino)-*N*-(pyrimidin-2-yl)benzenesulfonamide (14)

Yield, 65 %; m.p. 212.9 °C. IR: 3367, 3179 (NH), 3078 (arom.), 2937, 2870 (aliph.), 1635 (CO), 1577 (CN), 1375,1178 (SO_2_), 883 (CCl). ^1^HNMR: 3.4 [s, 3H, N-CH_3_], 6.2, 7.3 [2d, 2H, 2CH quinoline, *J* = 7.4 Hz], 6.6, 7.6 [2d, 2H, CH = CH, *J* = 7.5 Hz], 7.0–8.6 [m, 15H, Ar–H + SO_2_NH], 12.0 [s, 1H, NH]. ^13^CNMR: 40.5, 95.5, 100.3, 112.6 (2), 115.9, 116.0, 119.5, 120.2 (2), 125.8, 128.1 (2), 130.3 (2), 132.9, 133.7, 134.3, 134.6, 142.8, 144.3, 145.2, 146.9, 157.6 (2), 157.7, 158.6, 172.5, 186.6. MS m/z (%): 571 (M^+^) (33.2), 158 (100). Anal. Calcd. For C_29_H_23_ClN_6_O_3_S (571.05): C, 60.99; H, 4. 06; N, 14.72. Found: C, 61.28; H, 4.32; N, 14.47.

### 4-((*E*)-3-(4-((*E*)-7-chloro-1-methylquinolin-4(1*H*)-ylideneamino)phenyl)-3-oxoprop-1-enylamino)-*N*-(4-methylpyrimidin-2-yl)benzenesulfonamide (15)

Yield, 78 %; m.p. 274.8 °C. IR: 3366, 3259 (NH), 3076 (arom.), 2962, 2870 (aliph.), 1635 (CO), 1562 (CN), 1373, 1182 (SO_2_), 773 (CCl). ^1^HNMR: 2.3 [s, 3H, CH_3_], 3.4 [s, 3H, NCH_3_], 6.2, 7.6 [2d, 2H, 2CH quinoline, *J* = 7.3 Hz], 6.6, 7.3 [2d, 2H, CH = CH, *J* = 7.4 Hz], 7.5–8.5 [m, 13H, Ar–H], 10.3, 12.0 [2s, 2H, NH + SO_2_NH]. ^13^CNMR: 23.7, 40.5, 95.4, 100.2, 104.9, 112.4 (2), 114.9, 115.2, 115.8, 119.6 (2), 128.2, 129.5 (2), 130.5 (2), 132.9, 134.4, 134.6, 142.8, 144.3, 145.3, 146.7, 152.4, 157.4, 158.0, 168.6, 172.5, 186.6. MS m/z (%): 585 (M^+^) (9.36), 172 (100). Anal.Calcd. For C_30_H_25_ClN_6_O_3_S (585.08): C, 61.59; H, 4.31; N, 14.36. Found: C, 61.29; H, 4.59; N, 14.09.

### 4-((*E*)-3-(4-((*E*)-7-chloro-1-methylquinolin-4(1*H*)-ylideneamino)phenyl)-3-oxoprop-1-enylamino)-*N*-(4,6-dimethylpyrimidin-2-yl)benzenesulfonamide (16)

Yield, 91 %; m.p. 97.9 °C. IR: 3354, 3239 (NH), 3055 (arom.), 2947, 2861 (aliph.), 1635 (CO), 1593 (CN), 1371, 1180 (SO_2_), 864 (CCl). ^1^HNMR: 2.2 [s, 6H, 2CH_3_], 3.4 [s, 3H, NCH_3_], 5.8, 7.2 [2d, 2H, 2CH quinoline, *J* = 7.3 Hz], 6.6, 7.7 [2d, 2H, CH = CH, *J* = 7.5 Hz], 7.8–8.5 [m, 13H, Ar–H + SO_2_NH], 12.0 [s, 1H, NH]. ^13^CNMR: 23.4 (2), 40.2, 95.3, 100.1, 104.7, 112.3 (2), 113.8, 114.6, 115.4, 120.6 (2), 125.7, 129.4 (2), 130.8 (2), 132.9, 133.7, 134.8, 144.8, 145.0, 146.9, 157.1, 167.7, 167.8 (2), 172.7, 189.3. MS m/z (%): 599 (M^+^) (2.71), 109 (100). Anal. Calcd. For C_31_H_27_ClN_6_O_3_S (599.10): C, 62.15; H, 4. 54; N, 14.03. Found: C, 62.36; H, 4.19; N, 14.29.

### 4-((*E*)-3-(4-((*E*)-7-chloro-1-methylquinolin-4(1*H*)-ylideneamino)phenyl)-3-oxoprop-1-enylamino)-*N*-(5-methoxypyrimidin-2-yl)benzenesulfonamide (17)

Yield, 84 %; m.p. 264.5 °C. IR: 3396, 3221 (NH), 3101 (arom.), 2979, 2865 (aliph.), 1637 (CO), 1593 (CN), 1371, 1178 (SO_2_), 862 (CCl). ^1^HNMR: 3.4 [s, 3H, NCH_3_], 3.9 [s, 3H, OCH_3_], 5.9, 7.4 [2d, 2H, 2CH pyrimidine, *J* = 7.1 Hz], 6.2, 7.3 [2d, 2H, 2CH quinoline, *J* = 7.8 Hz], 6.6, 7.6 [2d, 2H, CH = CH, *J* = 7.4 Hz], 7.7–8.6 [m, 11H, Ar–H], 10.3, 12.0 [2s, 2H, NH + SO_2_NH]. ^13^CNMR: 40.5, 56.7, 95.4, 100.2, 105.0 (2), 112.6, 115.1, 116.0, 119.6 (2), 125.8, 128.2 (2), 129.8 (2), 130.1, 133.7, 134.6, 142.8, 144.2, 144.9, 145.3, 149.9, 151.7, 152.4, 153.3, 172.5, 186.6, 186.9. MS m/z (%): 601 (M^+^) (11.87), 74 (100). Anal. Calcd. For C_30_H_25_ClN_6_O_4_S (601.08): C, 59.95; H, 4.19; N, 13.98. Found: C, 60.23; H, 3.81; N, 13.69.

### 4-((*E*)-3-(4-((*E*)-7-chloro-1-methylquinolin-4(1*H*)-ylideneamino)phenyl)-3-oxoprop-1-enylamino)-*N*-(2,6-dimethoxypyrimidin-4-yl)benzenesulfonamide (18)

Yield, 87 %; m.p. 232.6 °C. IR: 3387, 3201 (NH), 3097 (arom.), 2980, 2839 (aliph.), 1635 (CO), 1589 (CN), 1352, 1178 (SO_2_), 771 (CCl). ^1^HNMR: 3.4 [s, 3H, N-CH_3_], 3.7 [s, 6H, 2OCH_3_], 5.9 [s, 1H, CH pyrimidine], 6.2, 7.3 [2d, 2H, 2CH quinoline, *J* = 7.5 Hz], 6.6, 7.2 [2d, 2H, CH = CH, *J* = 7.8 Hz], 7.4–8.5 [m, 11H, Ar–H], 10.3, 12.0 [2s, 2H, NH + SO_2_NH]. ^13^CNMR: 40.5, 54.1, 54.9, 85.1, 95.6, 100.4, 104.9 (2), 115.4, 116.2, 119.5, 120.2 (2), 128.1, 129.8 (2), 132.7 (2), 132.9, 133.7, 134.6, 142.7, 144.2, 144.9, 145.2, 152.3, 160.8, 161.0, 164.7, 172.0, 186.6. MS m/z (%): 631 (M^+^) (34.47), 154 (100). Anal. Calcd. For C_31_H_27_ClN_6_O_5_S (631.10): C, 59.00; H, 4.31; N, 13.32. Found: C, 58.76; H, 4.62; N, 13.03.

### 4-((*E*)-3-(4-((*E*)-7-chloro-1-methylquinolin-4(1*H*)-ylideneamino)phenyl)-3-oxoprop-1-enylamino)-*N*-(5,6-dimethoxypyrimidin-4-yl)benzenesulfonamide (19)

Yield, 83 %; m.p. 110.5 °C. IR: 3365, 3230 (NH), 3095 (arom.), 2941, 2863 (aliph.), 1635 (CO), 1577 (CN), 1375, 1159 (SO_2_), 773 (CCl). ^1^HNMR: 3.4 [s, 3H, N-CH_3_], 3.6, 3.8 [2s, 6H, 2OCH_3_], 6.2, 7.2 [2d, 2H, 2CH quinoline, *J* = 7.6 Hz], 6.6, 7.6 [2d, 2H, CH = CH, *J* = 7.7 Hz], 7.7–8.4 [m, 11H, Ar–H], 8.5 [s, 1H, CH pyrimidine], 10.3, 12.0 [2s, 2H, NH + SO_2_NH]. ^13^CNMR: 40.5, 54.2, 56.5, 95.3, 100.1, 112.6 (2), 115.8, 119.4, 120.8 (2), 127.9, 129.5 (2), 130.2, 133.0 (2), 133.8, 134.7, 142.9, 144.7, 145.1, 146.9, 149.8, 150.9, 152.0, 154.3, 161.7, 172.5, 186.6. MS m/z (%): 631 (M^+^) (22.13), 189 (100). Anal. Calcd. For C_31_H_27_ClN_6_O_5_S (631.10): C, 59.00; H, 4.31; N, 13.32. Found: C, 59.31; H, 4.04; N, 13.10.

### 4-((*E*)-3-(4-((*E*)-7-chloro-1-methylquinolin-4(1*H*)-ylideneamino)phenyl)-3-oxoprop-1-enylamino)-*N*-(1*H*-indazol-6-yl)benzenesulfonamide (20)

Yield, 89 %; m.p. 100.1 °C. IR: 3374, 3231 (NH), 3086 (arom.), 2978, 2848 (aliph.), 1635 (CO), 1589 (CN), 1363, 1151 (SO_2_), 819 (CCl). ^1^HNMR: 3.4 [s, 3H, N-CH_3_], 5.8, 6.6 [2d, 2H, 2CH quinoline, *J* = 7.2 Hz], 6.2, 6.8 [2d, 2H, CH = CH, *J* = 7.5 Hz], 7.0–8.5 [m, 16H, Ar–H + SO_2_NH], 10.8, 12.0 [2s, 2H, 2NH]. ^13^CNMR: 40.5, 91.1, 95.5, 100.4, 113.0, 115.1 (2), 115.4, 116.3, 119.5, 119.6, 119.8, 120.0, 120.6, 125.8, 129.0 (2), 129.8 (2), 132.1, 132.8, 133.5, 137.3, 140.7, 143.6, 144.3, 145.3, 146.8, 147.0, 154.3, 173.4, 189.8. MS m/z (%): 609 (M^+^) (51.63), 117 (100). Anal. Calcd. For C_32_H_25_ClN_6_O_3_S (609.10): C, 63.10; H, 4.14; N, 13.80. Found: C, 62.76; H, 4.40; N, 14.18.

### 4-((*E*)-3-(4-((*E*)-7-chloro-1-methylquinolin-4(1*H*)-ylideneamino)phenyl)-3-oxoprop-1-enylamino)-*N*-(quinoxalin-2-yl)benzenesulfonamide (21)

Yield, 66 %; m.p. 209.9 °C. IR: 3334, 3212 (NH), 3064 (arom.), 2981, 2863 (aliph.), 1635 (CO), 1591 (CN), 1375, 1178 (SO_2_), 767 (CCl). ^1^HNMR: 3.4 [s, 3H, NCH_3_], 6.2, 7.3 [2d, 2H, 2CH quinoline, *J* = 7.0 Hz], 6.6, 7.2 [2d, 2H, CH = CH, *J* = 7.3 Hz], 7.5–8.6 [m, 16H, Ar–H], 10.3, 12.0 [2s, 2H, NH + SO_2_NH]. ^13^CNMR: 40.5, 95.5, 100.3, 112.7 (2), 115.1, 116.0, 119.5,120.2 (2), 125.1, 126.3, 127.2, 127.3, 129.1, 130.1 (2), 131.1 (2), 132.8, 133.0, 133.8, 134.7, 138.0, 138.1, 139.2, 140.3, 142.7, 144.3, 149.7, 152.1, 169.6, 186.7. MS m/z (%): 621 (M^+^) (10.76), 177 (100). Anal. Calcd. For C_33_H_25_ClN_6_O_3_S (621.11): C, 63.81; H, 4.06; N, 13.53. Found: C, 63.49; H, 4.34; N, 13.23.

### (*E*)-3-(4-(4-aminophenylsulfonyl)phenylamino)-1-(4-((*E*)-7-chloro-1-methylquinolin-4(1*H*)-ylideneamino)phenyl)prop-2-en-1-one (22)

Compound **4** (3.65gm, 0.01 mol) and dapson (2.48 g, 0.01 mol) was added into ethanol (10 mL) and acetic acid (5 mL). The reaction was refluxed for 9 h and the solid obtained while hot was recrystallized from dioxane to give **22**.

Yield, 69 %; m.p. 95.2 °C. IR: 3446, 3348, 3213 (NH_2_, NH), 3100 (arom.), 2956, 2838 (aliph.), 1635 (CO), 1591 (CN), 1369, 1180 (SO_2_), 821 (CCl). ^1^HNMR: 3.4 [s, 3H, NCH_3_], 5.9 [s, 2H, NH_2_], 6.1, 7.4 [2d, 2H, 2CH quinoline, *J* = 7.8 Hz], 6.5, 6.6 [2d, 2H, CH = CH, *J* = 7.9 Hz], 7.5–8.6 [m, 15H, Ar–H], 12.0 [s, 1H, NH]. ^13^CNMR: 40.5, 95.5, 100.3, 113.3 (2), 113.4, 115.8 (2), 116.6, 119.3, 125.8 (2), 128.9 (4), 129.6 (2), 132.9 (3), 133.7, 135.9, 142.8, 144.2, 145.2, 146.9, 152.4, 154.3, 172.5, 186.6. MS m/z (%): 569 (M^+^) (19.87), 90 (100). Anal. Calcd. For C_31_H_25_ClN_4_O_3_S (569.07): C, 65.43; H, 4.43; N, 9.85. Found: C, 65.13; H, 4.71; N, 9.57.

### (2*E*,2′*E*)-3,3′-(4,4′-sulfonylbis(4,1-phenylene)bis(azanediyl))bis(1-(4-((*E*)-7-chloro-1-methylquinolin-4(1*H*)-ylideneamino)phenyl)prop-2-en-1-one) (23)

Compound **4** (7.30 gm, 0.02 mol) and Dapson (2.48 g, 0.01 mol) was added into ethanol (20 mL) containing acetic acid (10 mL). Reaction was refluxed for 12 h and the solid obtained while hot was recrystallized from acetic acid to give **23**.

Yield, 60 %; m.p. 186.9 °C. IR: 3143 (NH), 3078 (arom.), 2964, 2842 (aliph.), 1635 (CO), 1570 (CN), 1375, 1180 (SO_2_), 819 (CCl). ^1^HNMR: 3.4 [s, 6H, 2N-CH_3_], 6.2, 7.3 [2d, 4H, 4CH quinoline, *J* = 7.7 Hz], 6.6, 7.2 [2d, 4H, 2CH = CH, *J* = 7.8 Hz], 7.4–8.5 [m, 22H, Ar–H], 9.3, 12.0 [2s, 2H, 2NH]. ^13^CNMR: 40.5 (2), 95.8 (2), 100.7 (2), 104.9 (2), 113.4 (4), 115.8 (2), 116.7 (2), 119.6 (4), 125.8 (4), 129.7 (4), 132.8 (4), 133.6 (2), 134.6 (2), 142.6 (2), 144.0 (2), 145.9 (2), 146.7 (2), 152.3 (2), 172.5 (2), 186.7. MS m/z (%): 889 (M^+^) (6.48), 272 (100). Anal. Calcd. For C_50_H_38_Cl_2_N_6_O_4_S (889.85): C, 67.49; H, 4.30; N, 9.44. Found: C, 67.83; H, 4.66; N, 9.12.

### Anticancer screening

The cytotoxic activity in vitro of the novel synthesized compounds was measured using the sulforhodamine B stain (SRB) assay and the method of Skehan et al. [29]. The in vitro anticancer screening was done at pharmacognosy Department, College of Pharmacy, King Saud University, Riyadh, Saudi Arabia. Cells were plated in 96-multiwell plate (10^4^ cells/well) for 24 h before treatment with the compound(s) to allow attachment of cell to the wall of the plate. Test compounds were dissolved in dimethylsulfoxide. Different concentrations of the compound under test (10, 25, 50, and 100 μΜ) were added to the cell monolayer. Triplicate wells were prepared for each individual concentration. Monolayer cells were incubated with the compound(s) for 48 h at 37 °C and in an atmosphere of 5 % CO_2_. After 48 h, cells were fixed, washed and stained for 30 min with 0.4 % (Wt/vol) SRB dissolved in 1 % acetic acid. Excess unbound dye was removed by four washes with 1 % acetic acid and attached stain was recovered with Trise-EDTA buffer. Color intensity was measured using an enzyme-linked immunosorbent assay ELISA reader. Optical density was read at 510 nm. The relation between the surviving fraction and drug concentration was plotted to get the survival curve after the specified time The molar concentration required for 50 % inhibition of cell viability (*IC*_*50*_) was calculated and compared to the reference drug 2′,7′-dichlorofluorescein (DCF). The results are given in Table 1.

### Molecular docking

“All the molecular modeling studies were carried out on an Intel Pentium 1.6 GHz processor, 512 MB memory with Windows XP operating system using Molecular Operating Environment (MOE, 10.2008) software. All the minimizations were performed with MOE until a RMSD gradient of 0.05 kcal mol^−1^ Å^−1^ with MMFF94X force field and the partial charges were automatically calculated. The protein data bank file (PDB: 3S2A) was selected for this purpose. The file contains PI3K enzyme co-crystallized with a quinoline ligand obtained from protein data bank. The enzyme was prepared for docking studies where: (i) Ligand molecule was removed from the enzyme active site. (ii) Hydrogen atoms were added to the structure with their standard geometry. (iii) MOE Alpha Site Finder was used for the active sites search in the enzyme structure and dummy atoms were created from the obtained alpha spheres. (iv) The obtained model was then used in predicting the ligand enzymes interactions at the active site”.

## Conclusion

In summary, we had synthesized a novel series of benzene-sulfonamide derivatives. Seven products **1**, **2**, **4**, **7**, **11**, **14** and **17** presented sound anticancer activity hostile to lung (A594 Raw), hela, and colorectal (lovo) cancer cell lines with better or comparable activity to DCF. Moreover, molecular docking for these active compounds showed proper fitting on the active site of PI3K enzyme suggesting their action as inhibitors for this enzyme but more investigation should be carried out in the future to explore precisely the mechanism of the action of the synthesized derivatives.
